# Factors influencing nutrition literacy in patients of colorectal cancer: a cross-sectional study

**DOI:** 10.3389/fnut.2025.1526388

**Published:** 2025-03-04

**Authors:** Jing Zhang, Dan Li, Jiai Yan, Ju Yang, Jing Sun, Yiran Liu, Yanping Xia, Hong Cao, Jiao Hua, Feng Zhang, Yingyu Wang

**Affiliations:** ^1^Nutritional Department, Affiliated Hospital of Jiangnan University, Wuxi, China; ^2^Wuxi Medical College, Jiangnan University, Wuxi, China; ^3^Yixing Institute of Food and Biotechnology Co., Ltd., Yixing, China

**Keywords:** nutrition literacy, colorectal cancer, influencing factors, quality of life, nutritional education

## Abstract

**Background:**

Colorectal cancer (CRC) patients often experience varying degrees of malnutrition both pre-and post-treatment, highlighting the importance of their nutritional knowledge. However, studies on nutrition literacy (NL) in this population remain scarce. This study aims to evaluate the level of NL in colorectal cancer patients and identify key factors influencing NL.

**Methods:**

A total of 245 colorectal cancer patients participated in this study. The questionnaire included five sections: sociodemographic information, the Chinese Version of the Nutrition Literacy Assessment Instrument (CHI-NLit), the Montreal Cognitive Assessment Scale (MoCA), and the Hospital Anxiety and Depression Scale (HADS). Both univariate and multivariate analyses were performed to examine sociodemographic determinants of NL. We used Pearson and Spearman correlation coefficients to assess relationships between NL, MoCA and HADS.

**Results:**

The overall NL level among CRC patients was moderately low, with an average score of 19.224 ± 4.391—significantly below the normative neutrophil score of 21.5. Among the assessed dimensions, food groups received the lowest scores while food label calculation achieved the highest. Significant predictors influencing NL levels included age, years of education, family annual income, in review duration of illness, number of hospitalizations, memory and attention abilities and anxiety and depress symptoms.

**Conclusion:**

This study provides a comprehensive examination of NL in CRC patients. The findings indicate a relatively low level of NL within this group. Younger age, higher income levels, and urban residency correlated positively with elevated NL. Factors such as illness duration, number of hospitalizations, cognitive function measured by relevant scales are also emerged as significant determinants impacting NL. To enrich the research on NL, it is essential to conduct further data collection. From a clinical perspective, this evidence-based framework enables the development of stratified nutritional intervention protocols, specifically targeting vulnerable subgroups (e.g., elderly patients, rural dwellers, and those with extended illness duration). Such precision approaches hold significant potential to optimize dietary adherence, mitigate treatment-related complications, and ultimately enhance long-term quality of life in cancer survivorship care.

## 1 Introduction

Colorectal cancer (CRC) is the third most commonly diagnosed cancer worldwide and the second leading cause of cancer death in China (1). Diet plays an important role in CRC patients (2) and can affect the nutritional status of patients. During the course of treatment, patients frequently encounter a range of nutritional challenges and experience notable changes in their dietary habits (3). A survey reveals that more than 50% of patients report a loss of appetite during chemotherapy (4), which not only hinders physical recovery but also significantly impacts their quality of life (3).

Nutrition Literacy (NL) is derived from Health Literacy and refers to an individual’s capacity to acquire, process, and comprehend nutritional information, as well as the skills required to make informed nutritional decisions. NL represents a specialized form of Health Literacy (5, 6). It refers to understanding and applying healthy nutrition practices and plays a crucial role in determining eating behavior (7). There are various definitions and concepts of NL. NL encompasses six dimensions (8) knowledge, understanding, obtaining skills, applying skills, interactive skills, and critical skills (9). Individuals with high levels of NL adhere to dietary guidelines to make healthy food choices (10–12). NL has been identified as key components in the promotion and maintenance of healthy dietary practices (11, 13). However, research on NL remains limited. In recent years, scholars have devoted attention to developing and validating NL measurement instruments tailored for diverse populations, as well as conducting cross-sectional studies among these groups (14–18). This study will employ the NL assessment tool originally developed by Gibbs et al. (19) in 2012 for chronic disease patients and subsequently adapted and culturally validated by Chen in 2020 (20). The tool has been optimized to align with Chinese dietary practices. This study aims to assess the NL levels of CRC patients through questionnaire survey and to explore the influencing factors of these levels.

Since 2016, the State Council has successively issued key documents including “The Medium and Long-Term Plan for the Prevention and Control of Chronic Diseases in China (2017–2025)” (21) and “The Outline of the ‘Healthy China 2030’ Plan” (22). These policies aim to enhance the overall nutritional health status of the population and have incorporated “residents’ nutrition literacy levels” as one of the primary indicators for the development of Healthy China. CRC is a chronic disease that is closely associated with nutritional status. The NL of patients with CRC appears to be significantly correlated with their overall health outcomes. However, there remains a notable gap in systematic research examining the NL levels and influencing factors specific to this patient population. Given the limited research on NL, current interventions focus on diversified education models (23, 24), family-empowerment programs (25), and digital health technology (26). However, there are no specific intervention plans formulated based on the influencing factors of NL. Therefore, the main objective of this study was to assess the level of NL in CRC patients and to explore the factors affecting NL in CRC patients using a cross-sectional study design. This research aims to enrich the content of nutrition education, provide a theoretical foundation for nutrition literacy interventions, and offer scientific evidence to support the expansion of nutrition care methods.

## 2 Materials and methods

### 2.1 Study population and sampling criteria

A cross-sectional correlational study was conducted from April to July 2024 in Jiangsu Province, China. This study focuses on patients diagnosed with colon or rectal cancer who are preoperative, postoperative, or undergoing radiotherapy and chemotherapy. Participants were recruited from the inpatient wards of the Gastroenterology, Gastrointestinal Surgery, and Oncology departments at Jiangnan University Affiliated Hospital. Participants in this study were CRC patients aged 18 to 75, with intact cognitive function and the ability to independently complete questionnaires. They voluntarily consented to participate in the study. Data collection was conducted through face-to-face administration of questionnaires. Questionnaires were deemed complete if they satisfied the following criteria: no more than one-third of the questionnaire items had missing data, and the questionnaire successfully passed the deception detection test. This rephrasing maintains the original meaning while altering the structure and wording to reduce similarity with the original text. The study excluded individuals with neurological or psychiatric disorders or incomplete data to ensure the accuracy of the results. In addition, participants who expressed disinterest or followed special diets were randomly replaced to maintain the integrity of the study. According to the method of calculating the sample size of impact factor analysis, it is generally considered that the sample size should be taken as 5 to 10 times the number of variables, and there are 31 variables in this study, so the sample content of this study is a minimum of 155 cases and a maximum of 310 cases. To further refine the determination of the sample size, the pre-survey results showed that *p* = 40%. The sample size is calculated by the following formula: *n* = t^2^PQ/d^2^ = 225 (*α* = 0.05, *t* = 1.5, *p* = 0.39, *q* = 1 - *p* = 0.6, *d* = 0.1 × *p* = 0.04). Considering the 20% sample failure rate, the final sample was determined to be 245 cases.

### 2.2 Main variables

For the specific objectives of this study, the following variables were utilized: general demographic factors including gender, age, years of education, occupation, current residence, primary caregiver, and annual household income; as well as disease-related risk factors such as tumor location and stage, chronic disease status, current treatments, duration of illness, total number of hospitalizations, and smoking and drinking habits.

### 2.3 Assessment of NL

NL was assessed using the Chinese Version of the Nutrition Literacy Assessment Instrument (CHI-NLit), which was validated in the Chinese version in 2019. The CHI-NLit consists of 6 dimensions (Nutrition and health dimension; The source of energy in food dimension; Household food Measurement dimension; Food label calculation dimension; Food groups dimension and Consumer skill dimension) and 38 items (20), each of which is scored in a standardized multiple-choice format with four choices. Each item is in the form of a standardized multiple-choice question with four choices, with one point for a correct answer and zero points for an unanswered or incorrect answer. The score of the total scale ranges from 0 to 38 points, with a score of 21.5 as the cut-off value for dividing the lower and normal levels of NL (27). When the score is <21.5, it indicates that the patient’s NL level is insufficient, and when the score is >21.5, it indicates that the patient’s NL level is normal.

### 2.4 Assessment of cognitive function

The Montreal Cognitive Assessment Scale (MoCA) (28) was employed to evaluate the cognitive functioning of the patients. This scale assesses various cognitive domains, including attention and concentration, executive function, memory, language, visuospatial skills, abstract thinking, as well as computation and orientation. The total score for this scale is 30 points; an additional point is awarded if the subject has 12 years or fewer of education. A score around 16 indicates a diagnosis of Alzheimer’s disease; a score near 22 suggests mild cognitive impairment; scores of 26 or higher are classified as normal; while a perfect score is 30.

### 2.5 Assessment of anxiety depression symptom

Hospital Anxiety and depression scale (HADS) was revised by Zigmond et al. (29). It is divided into two subscales, anxiety and depression, with a total of 14 entries, of which there are 7 entries in the anxiety assessment part and 7 entries in the depression assessment part, the highest score of the subscale is 21 points, and the lowest score is 0 points, the higher the score is The higher the score, the higher the likelihood of anxiety and depression. The higher the score, the greater the likelihood of anxiety and depression. Scale scoring criteria: 0–7 is asymptomatic, 8–10 is likely to be symptomatic, and ≥ 11 is definitely anxious and depressed (30).

### 2.6 Statistical analysis

The recovered data were assigned unique identification numbers and entered into the database using EpiData 3.1. All statistical analyses were conducted using IBM SPSS Statistics 27.0 Percentage, frequency, mean, standard deviation, and a confidence level of 0.95 were used to describe study variables. Pearson’s correlation coefficient, independent samples t-test and one-way ANOVA were used to determine the relationship between the variables and the level of NL. A *p*-value ≤ 0.05 was considered statistically significant, and a *p*-value ≤ 0.001 was considered highly significant. For the analysis, when the independent variable is categorical and the dependent variable is normally distributed with homogeneity of variance, a t-test is employed for comparisons between two groups, while one-way ANOVA is used for comparisons among multiple groups. Non-parametric tests are utilized if the dependent variable deviates from normality or exhibits heterogeneity of variance. When the independent variable is continuous, Pearson correlation analysis is applied for normally distributed data, whereas Spearman rank correlation analysis is used for non-normally distributed data. For multivariate analysis, logistic regression is conducted. Figure was drawn with GraphPad Prism Version 9.0.0.

## 3 Results

### 3.1 Demographic and clinical characteristics

Two hundred forty-five CRC patients (130 males, 115 females) participated in the study. The mean age of samples was 60.224 ± 9.435. Overall, more than half of the participants were in tumor stage I-II (54.7%). The majority of participants had at least one other chronic disease (84.1%) and experienced 2–5 total hospitalizations (80%). Additional detailed socio-demographic information about the participants, as well as clinical disease characteristics, are presented in Tables 1, 2.

**Table 1:** Levels of NL based on different baseline characteristics.

Characteristic	Overall (*n* = 245)	NL levels *n* (%)		
		Insufficient	Normal	t or F, p
Age, median (IQR)	63 (55;67)			23.667, **<0.001**
<50 years old, *n* (%)	32 (13.1%)	11 (34.4%)	21 (65.6%)	
50–59 years old, *n* (%)	78 (31.8%)	41 (52.6%)	37 (47.4%)	
>60 years old, *n* (%)	135 (55.1%)	116 (85.9%)	19 (14.1%)	
Gender, *n* (%)	245 (100%)	165 (67.3%)	80 (32.7%)	0.029, 0.866
Male	130 (53.1%)	103 (79.2%)	27 (20.8%)	
Female	115 (46.9%)	88 (76.5%)	27 (23.5%)	
Years of education, median (IQR)	9 (6;10)			55.584, **<0.001**
<6 years, *n* (%)	122 (49.8%)	115 (94.3%)	7 (5.7%)	
6–8 years, *n* (%)	58 (23.7%)	38 (65.5%)	20 (34.5%)	
9–11 years, *n* (%)	44 (18.0%)	11 (25%)	33 (75%)	
12–16 years, *n* (%)	21 (8.5%)	4 (19.1%)	17 (80.9%)	
Occupation, *n* (%)				22.399, **<0.001**
Retirement	170 (69.4%)	134 (78.8%)	36 (21.2%)	
Brain worker	33 (11.8%)	10 (30.3%)	23 (69.7%)	
Non-manual laborer	42 (18%)	24 (57.1%)	18 (42.9%)	
Residence, *n* (%)				
City	130 (53.1%)	76 (58.5%)	54 (41.5%)	5.073, **<0.001**
Village	115 (46.9%)	92 (80%)	23 (20%)	
Primary caregiver, *n* (%)				0.974, 0.379
Spouse	165 (67.3%)	114 (69.1%)	51 (30.9%)	
Sons and daughters	39 (15.9%)	27 (69.2%)	12 (30.8%)	
Others	41 (16.8%)	27 (65.9%)	14 (34.1%)	
Household income/yr, *n* (%)				
<20,000RMB	47 (19.2%)	38 (80.8%)	9 (19.2%)	
20,000–100,000RMB	164 (66.9%)	114 (69.5%)	50 (30.5%)	
>100,000RMB	34 (13.9%)	16 (47.1%)	18 (52.9%)	6.360, **0.002**

The bold values in this table represent the *p*-values of the variables that exhibit statistically significant differences.

**Table 2:** Levels of NL based on different clinical and tumor characteristics.

Characteristics	Overall (*n* = 245)	NL levels *n* (%)		
		Insufficient	Normal	t or F, p
Type of Cancer, *n* (%)				0.029, 0.972
Colon cancer	73 (29.8%)	50 (68.5%)	23 (31.5%)	
Rectal cancer	128 (52.2%)	85 (66.4%)	43 (33.6%)	
Colorectal cancer	44 (18.0%)	33 (75%)	11 (25%)	
Tumor stage, *n* (%)				0.453, 0.637
Stage 0	69 (28.2%)	49 (71.0%)	20 (29.0%)	
Stage I-II	134 (54.7%)	93 (69.4%)	41 (30.6%)	
Stage III-IV	42 (17.1%)	26 (61.9%)	16 (38.1%)	
Chronic diseases, *n* (%)				
No	39 (15.9%)	30 (76.9%)	9 (23.2%)	2.380, 0.095
One	161 (65.7%)	108 (67.1%)	53 (32.9%)	
More than one	45 (18.4%)	30 (66.7%)	15 (33.3%)	
Current treatments, *n* (%)				0.119, 0.949
None	43 (17.6%)	29 (70.7%)	12 (29.3%)	
Surgery	143 (58.4%)	97 (67.8%)	46 (32.17%)	
Radiotherapy	41 (16.7%)	30 (73.2%)	11 (26.8%)	
Chemoradiotherapy	20 (8.3%)	12 (60%)	8 (40%)	
Duration of illness, *n* (%)				
<1 years	56 (22.9%)	39 (69.6%)	17 (30.4%)	2.929, **0.034**
2–5 years	133 (54.3%)	97 (72.9%)	36 (27.1%)	
>5 years	34 (13.8%)	20 (58.8%)	14 (41.2%)	
Number of hospitalizations, *n* (%)				5.113, **0.002**
1times	32 (13.1%)	26 (81.3%)	6 (18.7%)	
2–3 times	114 (46.5%)	81 (71.1%)	33 (28.9%)	
4–5 times	82 (33.5%)	55 (67.1%)	27 (32.9%)	
>5 times	17 (6.9%)	6 (35.3%)	11 (64.7%)	
Smoking/drinking, *n* (%)				0.102, 0.959
None	27 (11.0%)	18 (66.7%)	9 (33.3%)	
Smoking	120 (49.0%)	81 (67.5%)	39 (32.5%)	
Drinking	77 (31.4%)	52 (67.5%)	25 (32.5%)	
Both	21 (8.6%)	17 (81%)	4 (19%)	

The bold values in this table represent the *p*-values of the variables that exhibit statistically significant differences.

### 3.2 Univariate analysis influencing the level of NL in CRC patients

**Figure 1:**
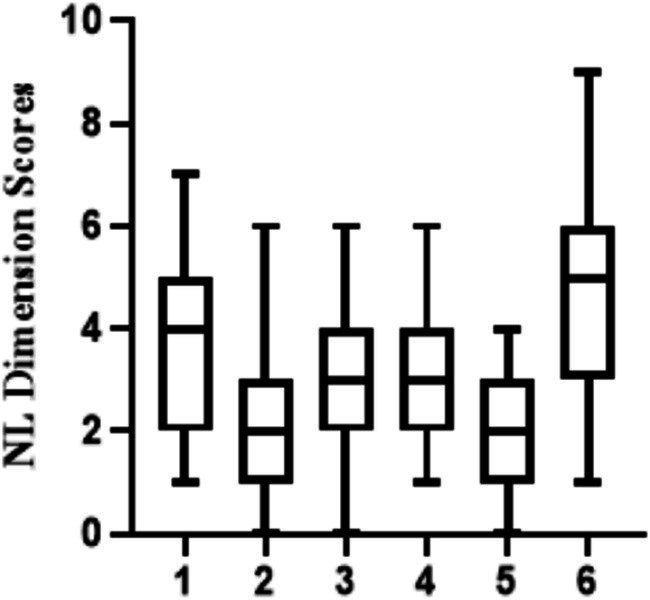
Median of NL scores of each scale as assessed by CHI-NLit. Higher scores indicate higher NL levels (error bars ≙ ranges). CHI-Nlit questionnaire; dimension 1: Nutrition and health; dimension 2: The source of energy in food; dimension 3: Household food Measurement; dimension 4: Food label calculation; dimension 5: Food groups; dimension 6: Consumer skill.

In this study, the highest score of NL level was 32 and the lowest score was 8. The average score of the NL was19.224 ± 4.391. Among the dimensions of NL, the dimension with the lowest score was food groups, which is means to interactive skills and the dimension with the highest score was food label calculation, which is means to applying skills. A total of 77 patients (31.4%) had a NL level score greater than the cutoff value of 21.5, which was basically at the lower middle level. Detailed results of all subscales are displayed in Figure 1.

In Tables 1, 2, the sociodemographic characteristics and Clinical characteristics were reported in comparison to the level of NL. There is no difference in the level of NL between males and females. The study indicated that patients who were younger, more highly educated, residing in urban areas, brainworkers, and with greater household incomes demonstrated a higher level of NL. Furthermore, the study revealed a correlation between the level of NL and several key factors, including the duration of the illness, and the total number of hospitalizations.

Then, we correlated the CHI-NLit score with the MoCA, and HADS. The coefficient of correlation between the scores is shown in Table 3. We found that NL scores were positively associated with cognitive function, and negatively associated with anxiety.

**Table 3:** Participants’ scores on other scales and correlations with NL scores.

	Pearson related values	Spearman related values	p
MoCA			
Total score		**0.158**	**0.013**
Visuospatial/Executive		0.058	0.364
Naming		0.117	0.067
Attention		**0.128**	**0.045**
Language		0.087	0.174
Abstraction		−0.025	0.697
Memory		**0.163**	**0.011**
Orientation		−0.001	0.993
HADS			
Anxiety	−0.904		**<0.001**
Depression		0.133	**0.038**

The bold values in this table represent the *p*-values of the variables that exhibit statistically significant differences.

### 3.3 Multifactorial analysis of the factors affecting the level of NL in CRC patients

The results of the univariate analysis were used to inform the binary logistic regression forward stepwise selection, which was conducted to analyze the factors affecting the level of NL in CRC patients. The entry level for this analysis was set at *α* = 0.05, with *β* = 0.10 used as the exclusion level. The findings revealed that: (1) The anxiety dimension in the anxiety and depression scale was identified as a risk factor for the level of patient NL. It was found that as anxiety levels increased, NL levels decreased. And this implies that for every one-unit increase in anxiety level, the NL level decreases by 0.218 times. (2) The cognitive function scales’ attract dimension was identified as a statistically significant predictor in the model, indicating that lower levels of this dimension were associated with lower levels of NL. This means that for every one-unit increase in the Attraction dimension score of cognitive function, the level of NL will increase by 9.049 times. Detailed results of all subscales are displayed in Table 4.

**Table 4:** Logistic regression analysis of factors influencing the level of NL.

Factors	Coefficient of regression β	Standard error	Wald***χ***^2^	P	OR	95%CI
Variables	10.862	4.346	6.246	0.012	52165.328	
Anxiety and depression (anxiety)	−0.1.523	0.393	15.057	<0.001	0.218	0.101–0.471
MoCA (Abstraction)	2.203	0.507	18.857	<0.001	9.049	3.348–24.453

## 4 Discussion

Our study conducted at Jiangnan University Hospital in Wuxi, China, analyzed the NL level of 245 CRC patients. The objective of this study is to evaluate NL levels of patients and identify the factors that influence these levels. The response rate was 92.3% due to detailed information provided by researchers. The results demonstrated that only 31.4% of participants exhibited a high level of NL, emphasizing the importance of enhancing patients’ nutritional knowledge during and after cancer treatment. In the food label calculation domain, CRC patients showed high levels. However, in the food groups domain, CRC patients exhibited low levels. This indicates that CRC patients acquire general nutritional knowledge through routine inpatient nutrition education; however, they struggle to comprehend more specialized information, such as precise energy intake and the content of food-related components. People focus mainly on nutritional knowledge but do not make good use of this knowledge in their lives, such as how to use nutritional labels to judge the nutritional value of food. This phenomenon has also been observed among healthcare professionals (31). This underscores the necessity for healthcare professionals to offer CRC patients a more comprehensive and personalized nutritional regimen that empowers them to evaluate and integrate complex nutritional information while making informed decisions (32) (see Table 4).

NL is closely related to social factors (33). Both internal and external factors may affect the acquisition and utilization of nutritional knowledge (34). In this study, we recruited 53.1% men and 46.1% women with CRC consistent with previous research indicating a higher incidence of CRC among men (35). In previous studies (36–39), it is not difficult to find that females show an advantage in NL. This observation may be attributed to the fact that women tend to exhibit greater attentiveness to their dietary habits in daily life. However, in our study, gender factors did not show significant differences (*p* = 0.672), which may be related to the fact that the patients recruited were mostly middle-aged and elderly. With age, women are less influenced by social and cultural factors to a greater extent, and therefore pay more attention to appearance and health, so the importance of diet and nutrition has not significantly increased. There is a close relationship between educational level and NL (40). Individuals with higher education levels tend to be more health-conscious, including in their diet and nutrition. Higher medical education usually includes nutrition courses, enabling CRC patients with higher education levels to better understand the importance of nutrition both theoretically and in practical application. Such courses often cover various aspects, including the functions of NL, the establishment of healthy eating habits, and the relationship between nutrition and diseases (17, 41). A longer duration of education is associated with higher levels of NL among patients, which facilitates the control and delay of disease progression. In this study, both place of residence and household income status significantly influence the level of NL among CRC patients. Those residing in urban areas with higher household incomes demonstrated elevated levels of NL, which is consistent with the findings of previous studies (42). Uneven regional economic development has led to disparities in the distribution of health knowledge resources, resulting in urban residents having greater access to channels and opportunities for acquiring essential nutritional information. Consequently, this contributes to significant variations in regional NL levels. NL can be considered as a modifiable risk factor of socioeconomic disparities in health. Enhancing the level of NL in the population or making health services more accessible to people with low NL may be a means to reach a greater equity in health (43). Meanwhile, the total number of hospitalizations also significantly influences the level of NL among CRC patients, with a greater frequency of hospitalizations correlating with higher levels of NL. This may be attributed to the multiple opportunities for nutritional education provided during hospitalization.

Research shows that NL comes from health literacy (6). Studies demonstrate that lower health literacy correlates with poorer health outcomes. This association is particularly evident among older adults and individuals with lower educational attainment. Additionally, reduced health literacy is linked to poorer self-rated health status. It further corresponds with decreased utilization of preventive health services. Higher rates of hospital admissions and mortality are also observed in this population. Finally, diminished physical and mental well-being has been consistently documented in these cases (44–49). In light of these findings, it seems plausible to suggest that the factors underpinning NL are similar to those associated with health literacy. Our study incorporated three key components. First, seven sociodemographic characteristics were analyzed: sex, age, educational attainment, occupation, residential location, primary caregiver, and annual household income. Second, seven disease-related risk factors were examined: tumor location and stage, chronic disease comorbidity, current therapeutic interventions, disease progression timeline, hospitalization frequency, and substance use patterns (tobacco and alcohol). Third, two validated assessment tools were utilized: the Montreal Cognitive Assessment (MoCA) and the Hospital Anxiety and Depression Scale (HADS). In other populations, such as college student, NL is related to their gender (50), birthplace, and nutrition education (51), which is similar to our study. This suggests that the factors influencing NL exhibit similar characteristics across diverse populations in relation to sociodemographic data. In this study, the highest score of NL level was 32 and the lowest score was 8. The mean score of NL was 19.224 ± 4.391, which is lower than the NL scores reported for patients with type 2 diabetes mellitus in a study by Chen in 2020. This finding indicates that caregivers should place greater emphasis on providing targeted health education and nutritional guidance. The results of univariate analysis showed that age, years of education, total annual household income, total number of hospitalizations, level of cognitive functioning and quality of life were correlated with the level of NL. This is consistent with other studies and health literacy studies (52). The evaluation results of CHI-NLit indicate that the level of nutritional knowledge is positively correlated with the “attention” dimension of cognitive function as assessed by MoCA, and negatively correlated with anxiety levels as excepted (52). Research indicates that, from a physiological perspective, several factors influence cognitive dysfunction in patients with CRC. These factors include age, sleep quality, the number of chemotherapy cycles, and post-chemotherapy nausea and vomiting (53). Our study has identified significant differences in cognitive function between elderly and younger patients. Healthcare providers should focus on identifying and addressing factors influencing cognitive impairment to improve cognitive function, enhance NL, and ultimately improve quality of life.

We also analyzed the interactions among research variables. Patients with higher education levels show better information processing abilities, particularly in attention, which enhances their understanding and application of nutritional knowledge. Our study found that cognitive function’s “attention” dimension is positively correlated with NL, while education level correlates with MoCA scores. This suggests that education indirectly improves NL by enhancing cognitive functions, especially attention. Additionally, NL is negatively correlated with anxiety but positively correlated with cognitive functions. Anxiety may impair knowledge absorption, reducing the positive impact of cognitive functions on NL. Frequent hospitalizations, which provide nutritional education, are positively correlated with NL, especially benefiting patients with higher cognitive functions. Future interventions for CRC patients with low NL should include personalized strategies: reinforcing nutritional education during hospitalization for less educated or rural patients using visual aids, and integrating psychological counseling with nutritional guidance for anxious patients to mitigate anxiety’s negative effects on NL.

The CHI-NLit scale was administered for the first time to CRC patients, which is one of the innovations of this study. This is also a good basis for rationalizing the use of measurement tools in the research. This is also the first study that links CRC patients with NL. Cross-sectional studies collect data at a specific point in time. If the sample does not adequately represent the target population, it may introduce selection bias. For instance, surveys limited to a particular region or specific group may lack generalizability. Additionally, cross-sectional studies cannot capture dynamic changes in population characteristics over time. For example, patients’ nutritional knowledge and literacy levels may vary across different treatment stages or changes in living environments, which cannot be reflected in a single-time-point survey. Therefore, future research should employ multi-time-point, multi-region, large-sample, and random sampling methods for both cross-sectional and longitudinal studies. Furthermore, other confounding variables (such as psychological state, gender, etc.) should be considered to enhance the reliability and validity of the conclusions. Future studies should further investigate NL intervention strategies and compare NL characteristics across different tumor types to enrich the existing body of NL research.

Improving the NL of CRC patients requires the mobilization of a variety of factors, and combining the mechanisms of each influencing factor on NL suggests that improving the literacy and overall quality of patients, developing individualized health education strategies, improving the social support system of patients, alleviating anxiety and depression, and doing a good job of cognitive screening and exercise are essential to improving the NL of patients. In summary, this study underscores the critical importance of NL in the management of CRC. The findings reveal a significant gap in nutritional knowledge among patients, which can adversely affect their treatment outcomes and quality of life. By addressing the factors that influence NL and implementing targeted educational interventions, healthcare providers can empower CRC patients to make informed dietary choices that support their health and well-being. Future research should continue to explore this vital area, ensuring that NL becomes a standard component of comprehensive cancer care.

## 5 Conclusion

This study provides a comprehensive examination of NL in CRC patients. The findings indicate a relatively low level of NL within this group. Younger age, higher income levels, and urban residency correlated positively with elevated NL. Factors such as illness duration, number of hospitalizations, cognitive function measured by relevant scales are also emerged as significant determinants impacting NL. To address the limitations of cross-sectional studies and improve the quality of future research on NL, we recommend expanding the sample scope by collecting data from diverse geographic regions and socio-economic backgrounds to ensure representativeness. Stratified sampling can help achieve balanced data across populations. Combining cross-sectional and longitudinal designs will capture dynamic changes over time, aiding in understanding causal relationships. Integrating quantitative and qualitative methods, such as interviews or focus groups, will provide deeper insights into respondents’ motivations, perspectives, and behaviors.

## Data Availability

The original contributions presented in the study are included in the article/supplementary material, further inquiries can be directed to the corresponding authors.
